# Amyloid-β, p-tau and reactive microglia are pathological correlates of MRI cortical atrophy in Alzheimer’s disease

**DOI:** 10.1093/braincomms/fcab281

**Published:** 2021-11-24

**Authors:** Irene Frigerio, Baayla D C Boon, Chen-Pei Lin, Yvon Galis-de Graaf, John Bol, Paolo Preziosa, Jos Twisk, Frederik Barkhof, Jeroen J M Hoozemans, Femke H Bouwman, Annemieke J M Rozemuller, Wilma D J van de Berg, Laura E Jonkman

**Affiliations:** 1 Section Clinical Neuroanatomy and Biobanking, Department of Anatomy and Neurosciences, Amsterdam UMC, Location VUmc, Amsterdam Neuroscience, Vrije Universiteit, 1081 HV Amsterdam, the Netherlands; 2 Department of Pathology, Amsterdam Neuroscience, Amsterdam UMC, Location VUmc, 1081 HV Amsterdam, the Netherlands; 3 Neuroimaging Research Unit, Division of Neuroscience, IRCCS San Raffaele Scientific Institute, 60-20132 Milan, Italy; 4 Neurology Unit, IRCCS San Raffaele Scientific Institute, 60-20132 Milan, Italy; 5 Department of Epidemiology and Biostatistics, Vrije Universiteit, 1081 HV Amsterdam, the Netherlands; 6 Department of Radiology and Nuclear Medicine, Amsterdam Neuroscience, Amsterdam UMC, Location VUmc, 1081 HV Amsterdam, the Netherlands; 7 Institutes of Neurology and Healthcare Engineering, University College London, London WC1E, UK; 8 Department of Neurology, Alzheimer Center Amsterdam, Amsterdam Neuroscience, Amsterdam UMC, Location VUmc, Alzheimer Centrum Amsterdam, 1081 HV Amsterdam, the Netherlands

**Keywords:** Alzheimer’s disease, cortical thickness, neuropathology, MRI, atypical Alzheimer’s disease

## Abstract

Alzheimer’s disease is characterized by cortical atrophy on MRI and abnormal depositions of amyloid-beta, phosphorylated-tau and inflammation pathologically. However, the relative contribution of these pathological hallmarks to cortical atrophy, a widely used MRI biomarker in Alzheimer’s disease, is yet to be defined. Therefore, the aim of this study was to identify the histopathological correlates of MRI cortical atrophy in Alzheimer’s disease donors, and its typical amnestic and atypical non-amnestic phenotypes. Nineteen Alzheimer’s disease (of which 10 typical and 9 atypical) and 10 non-neurological control brain donors underwent post-mortem *in situ* 3T 3D-T1, from which cortical thickness was calculated with Freesurfer. Upon subsequent autopsy, 12 cortical brain regions from the right hemisphere and 9 from the left hemisphere were dissected and immunostained for amyloid-beta, phosphorylated-tau and reactive microglia, and percentage area load was calculated for each marker using ImageJ. In addition, post-mortem MRI was compared to ante-mortem MRI of the same Alzheimer’s disease donors when available. MRI-pathology associations were assessed using linear mixed models. Higher amyloid-beta load weakly correlated with higher cortical thickness globally (*r* = 0.22, *P* = 0.022). Phosphorylated-tau strongly correlated with cortical atrophy in temporal and frontal regions (−0.76 < *r* < −1.00, all *P* < 0.05). Reactive microglia load strongly correlated with cortical atrophy in the parietal region (*r* = −0.94, *P* < 0.001). Moreover, post-mortem MRI scans showed high concordance with ante-mortem scans acquired <1 year before death. In conclusion, distinct histopathological markers differently correlated with cortical atrophy, highlighting their different roles in the neurodegenerative process, and therefore contributing to the understanding of the pathological underpinnings of MRI atrophic patterns in Alzheimer’s disease. In our cohort, no or only subtle differences were found in MRI-pathology associations in Alzheimer’s disease phenotypes, indicating that the histopathological correlates of cortical atrophy in typical and atypical phenotypes might be similar. Moreover, we show that post-mortem *in situ* MRI can be used as proxy for ante-mortem *in vivo* MRI.

## Teaser

Frigerio et al. report that in Alzheimer’s disease, amyloid-beta, phosphorylated-tau and reactive microglia load measured with immunohistochemistry, are important correlates of MRI cortical atrophy, acquired both ante-mortem *in vivo* and post-mortem *in situ*. In summary, distinct histopathological markers differently correlate with cortical atrophy, highlighting their different roles in the neurodegeneration process.

## Graphical Abstract

**Graphical Abstract:**
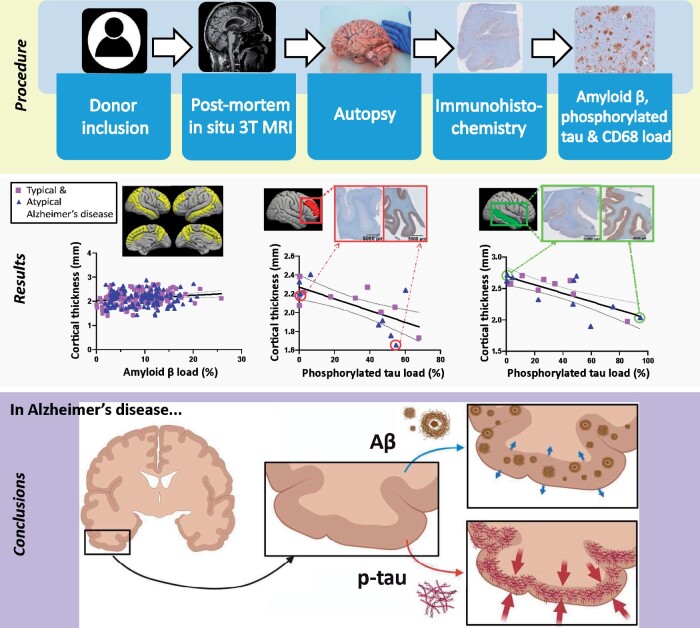

## Introduction

Alzheimer’s disease is a progressive neurodegenerative disease with a heterogeneous clinical presentation. Clinically, Alzheimer’s disease is defined as typical when memory deficits are the first complaints, and atypical when memory is initially spared while other symptoms are more prominent, such as visuospatial impairment, aphasia or behavioural/dysexecutive dysfunction.^1^ On MRI, typical Alzheimer’s disease is typically characterized by hippocampal and temporoparietal atrophy,^2^ whereas atypical presentations may show initial hippocampal sparing, and cortical atrophy in regions corresponding to clinical symptoms.^3^ Although hippocampal and temporoparietal atrophy are commonly used MRI biomarkers in Alzheimer’s disease, reliable imaging biomarkers ideally reflect disease state as well as underlying pathophysiological mechanisms in Alzheimer’s disease, and should be validated in a cohort of neuropathologically confirmed Alzheimer’s disease subjects.

Pathologically, Alzheimer’s disease is characterized by the accumulation of amyloid-beta (Aβ) plaques and phosphorylated-tau (p-tau) neurofibrillary tangles (NFT) in the grey matter.^4^^,^^5^ In addition, neuroinflammation plays a key role in Alzheimer’s disease.^6^^,^^7^ Reactive microglia tend to proliferate and increase with disease progression^6^^,^^7^ and seem to reflect p-tau deposition sites.^8^

Studies combining MRI and PET imaging as a proxy for pathology showed that the load and neuroanatomical distribution of tau tracers correlate with cortical atrophic patterns on MRI,^9–13^ while widespread cortical Aβ deposition^9^ does not correlate with cortical atrophy nor clinical presentation in clinically defined Alzheimer’s disease cases.^14^^,^^15^ The effect of reactive microglia load on cortical atrophy remains elusive, since the few studies that investigated its association with MRI atrophic patterns report contrasting results.^16^^,^^17^ Unfortunately, PET imaging has a limited resolution, and cannot evaluate different pathological processes together, but just one at each exam. As such, post-mortem histopathological examination remains the gold-standard to diagnose Alzheimer’s disease and to evaluate the combination of different markers.^18^

The aim of this study was to assess the association between post-mortem *insitu* (within the skull) MRI cortical thickness and histopathological hallmarks in clinically defined and pathologically confirmed Alzheimer’s disease subjects. Additionally, due to the clinical heterogeneity in Alzheimer’s disease, we explored the association between neuroanatomical distribution of MRI cortical atrophy and neuropathological patterns in clinically defined typical and atypical Alzheimer’s disease phenotypes.^19^ Lastly, we investigated the coherence between ante-mortem and post-mortem patterns in subsets of these patients. Results of this study will increase our knowledge on the pathophysiology of cortical atrophy, thereby contributing to the understanding of the pathological underpinnings of MRI atrophy patterns in Alzheimer’s disease.

## Materials and methods

Detailed methods are described in Supplementary material.

### Donor inclusion

In collaboration with the Netherlands Brain Bank (NBB; http://brainbank.nl, Accessed 14 October 2021), we included 19 Alzheimer’s disease donors from the Amsterdam Dementia Cohort.^20^ The Alzheimer’s disease donors could be further subdivided into 10 typical and 9 atypical Alzheimer’s disease donors based on clinical symptoms, of which 6 were diagnosed with the behavioural/dysexecutive variant,^3^ and 3 with posterior cortical atrophy.^21^ Neuropathological diagnosis was confirmed by an expert neuropathologist (A.R.) and performed according to the international guidelines of the Brain Net Europe II (BNE) consortium (http://www.brainnet-europe.org, Accessed 14 October 2021).^22^^,^^23^ Additionally, 10 age-matched pathologically confirmed non-neurological controls were selected from the Normal Aging Brain Collection Amsterdam (NABCA; http://nabca.eu, Accessed 14 October 2021).^24^ All donors signed an informed consent for brain donation, and the use of material and clinical information for research purposes. The procedures for brain tissue collection of NBB and NABCA have been approved by the Medical Ethical Committee of VUmc. For donor characteristics, see Supplementary Table 1.

### Post-mortem *in situ* and ante-mortem *in vivo* MRI acquisition

**Figure 1:**
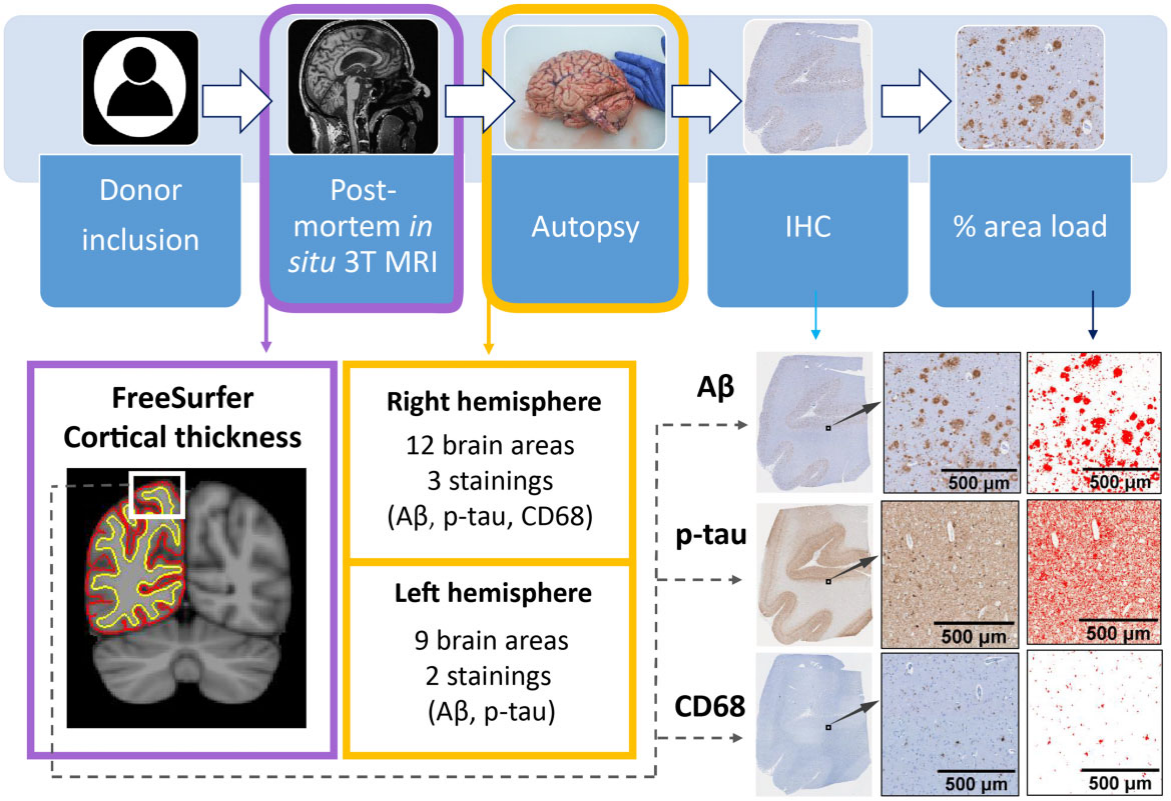
**Workflow of the post-mortem MRI-pathology pipeline.** Once the donors were included in the study, they received a post-mortem *in situ* 3T MRI, and cortical thickness was calculated with FreeSurfer^25^ from the 3D T1w image (purple box). After the MRI scan, autopsy was performed, and brain tissue was processed for immunohistochemistry against Aβ, p-tau and CD68 (yellow boxes), which were quantified using ImageJ. The correlation between cortical thickness and %area of immunoreactivity was investigated via linear mixed models (dashed grey arrow). Aβ, amyloid-beta; IHC, immunohistochemistry; p-tau, phosphorylated-tau.

Post-mortem 3T brain *insitu* MRI acquisition was acquired according to a previously described pipeline^24^ (for an overview of our workflow, see Fig. 1). Briefly, 3T MRI was acquired on a magnetic resonance scanner (Signa-MR750, General Electric Medical Systems, United States) with an eight-channel phased-array head-coil. The following post-mortem sequences were acquired for all subjects: (i) a sagittal 3D T_1_-weighted fast spoiled gradient echo sequence [repetition time (TR) = 7 ms, echo time (TE) = 3 ms, flip angle = 15°, 1-mm-thick axial slices, in-plane resolution= 1.0 × 1.0 mm^2^]; (ii) and a sagittal 3D fluid attenuation inversion recovery (FLAIR) sequence [TR = 8000 ms, TE = 130 ms, inversion time (TI) = 2000–2500 ms, 1.2-mm-thick axial slices, in-plane resolution= 1.11 × 1.11 mm^2^], with TI corrected for post-mortem delay. Moreover, 14 out of 19 Alzheimer’s disease cases included in our study had ante-mortem *invivo* 3T MRI scans available, and these were included for comparison with post-mortem MRI. Ante-mortem sequences that were acquired for diagnostics were retrospectively obtained from the Amsterdam Dementia Cohort^20^ from two different scanners (3T GE MR750 and Philips 3T Achieva). The parameters were slightly different between scanners, with TR varying between 7.8 and 7.9 ms, and TE between 2.9 and 5.2 ms, while voxel size was fixed at 1 mm^3^ (see Supplementary Table 2 for sequence details).

### Filling of white matter hyperintensities on MRI

Post-mortem T_1_-weighted images were lesion filled to reduce lesion effects on subsequent automated segmentations. Segmentation of white matter abnormalities was performed on FLAIR images using multi-view convolutional neural network with batch normalization followed by manual editing, obtaining lesion maps, which were registered to the 3D T_1_ images. The refilling of the lesions was done using LEsion Automated Preprocessing (LEAP).^26^

### MRI cortical thickness and brain volume assessment

Image processing was performed using Freesurfer, version 6.0 (http://surfer.nmr.mgh.harvard.edu, Accessed 14 October 2021).^25^ Images underwent inhomogeneity correction, removal of non-brain tissue, and segmentation into grey and white matter. Parcellation of the brain was done using the Desikan–Killany atlas.^27^ Cortical thickness was measured as the distance from the grey/white matter boundary to corresponding pial surface. The reconstructed datasets were visually inspected, and segmentation errors were corrected. Furthermore, post-mortem normalized brain volume, and normalized grey and white matter volumes were measured from the T_1_-weighted images using Structural Image Evaluation, using Normalisation, of Atrophy (SIENAX) (part of FSL 5.0.9; http://fsl.fmrib.ox.ac.uk/, Accessed 14 October 2021), which estimates brain tissue volume normalized for skull size.^28^

### Tissue sampling

Subsequent to MRI acquisition, the autopsy was performed, resulting in a total post-mortem delay within 10 hours for all brain donors. Formalin-fixed paraffin-embedded (4%, four weeks fixation) 12 tissue blocks from the following regions of the right hemisphere were used: superior and middle frontal gyrus, anterior and posterior cingulate gyrus, middle temporal gyrus, superior and inferior parietal gyrus, precuneus, occipital cortex (primary visual cortex) and hippocampus (including the entorhinal cortex, parahippocampal and fusiform gyrus as described before).^29^ Additionally, for 13 Alzheimer’s disease cases (7 typical and 6 atypical), 9 formalin-fixed paraffin-embedded (4%, 24–36 h fixation) tissue blocks from the left hemisphere were available from the same regions as described above, except for the hippocampus and posterior cingulate gyrus, adding the superior temporal gyrus (see Supplementary Table 3).

### Immunohistochemistry

6 µm sections from the above-mentioned regions were cut and mounted on superfrost+ glass slides (Thermo Scientific, USA). The sections from the right hemisphere were stained for Aβ (4G8), p-tau (AT8), and reactive microglia (CD68, clone KP1). The sections from the left hemisphere were additionally collected and stained for Aβ (4G8) and p-tau (AT8) (see Supplementary Table 4 for information on primary antibodies). Briefly, the sections were blocked for endogenous peroxidase using 0.3% hydrogen peroxide and 0.1% sodium azide in phosphate buffer saline (PBS; pH 7.4). The sections were immersed in 10 mM Citrate buffer pH 6.0 and heated to 120°C in an autoclave for antigen retrieval. Primary antibodies were diluted in normal antibody diluent (ImmunoLogic, Duiven, The Netherlands) and incubated overnight at 4°C. Primary antibodies were detected using EnVision (Dako, Glostrup, Denmark). Afterwards, antibodies were visualized using 3.3′-Diaminobenzidine (DAB, Dako) with Imidazole (50 mg DAB, 350 mg Imidazole and 30 µL of H_2_O_2_ per 100 mL of Tris-HCl 30 mM, pH 7.6). In between steps, PBS was used to wash the sections. After counterstaining with haematoxylin, the sections were dehydrated and mounted with Entellan (Merck, Darmstadt, Germany). To visualize the line of Gennari in the occipital cortex and therefore identify the striate area (i.e. primary visual cortex), we further performed a Kluver staining on these sections.

### Image analysis

Images were taken using a whole-slide scanner (Vectra Polaris, 20× objective) and quantified using Fiji ImageJ Version 1.52r (https://imagej.nih.gov/ij, Accessed 14 October 2021). Regions of interest containing all cortical layers were delineated in straight areas of the cortex to avoid over- or underestimation of pathology in sulci and gyri, respectively.^30^ After colour deconvolution, used to separate haematoxylin and DAB channels, the immunoreactivity of DAB staining was quantified using the auto-threshold plugin ‘maximum entropy’.^31^ The outcome measure was the % stained area per regions of interest of each section per immunohistochemistry marker.

### Statistics

Statistical analyses were performed in SPSS 26.0 (Chicago, IL). Normality was tested, and subsequently demographics between Alzheimer’s disease and controls, and between controls, typical and atypical phenotypes, were compared using parametric or non-parametric tests for continuous data, and Fisher exact test for categorical data. Cortical thickness between groups was tested with linear mixed models with age, gender and post-mortem delay as covariates, and Bonferroni *posthoc* correction. Pathological outcome measures were compared across groups with linear mixed models, using age and gender as covariates, and Bonferroni *posthoc* correction. The associations between cortical thickness and Thal phases/Braak NFT stages were calculated with Spearman’s correlation. Cortical thickness-pathology associations across regions were tested with linear mixed models in the Alzheimer’s disease and control group separately, and then within Alzheimer’s disease phenotypes with age, gender and post-mortem delay as covariates. For MRI-pathology associations across regions, we excluded the entorhinal cortex, as this region was significantly thicker than other brain areas (entorhinal cortical thickness: 3.29 mm ± 0.57, other areas: 2.20 mm ± 0.33, p < 0.001) and would drive the associations. To investigate the regional effect of each pathological marker on cortical thickness of each brain region, we used general linear models. To investigate the combined effect of all pathological markers on cortical thickness, we used linear regression analysis. Statistics at the brain area level were corrected for multiple comparisons using the false discovery rate approach (FDR),^32^ and the FDR corrected *P*-values were expressed as *q*-values.

### Data availability

The data that support the findings of this study are available from the corresponding author, upon reasonable request.

## Results

### Donor characteristics

Demographical, clinical, radiological and pathological data of Alzheimer’s disease and non-neurological control donors are summarized in Table 1. Age, disease duration and post-mortem delay did not differ between groups, whereas gender differed between controls and Alzheimer’s disease cases (*P* = 0.032). On MRI, normalized brain volume (−5.4% in Alzheimer’s disease compared to controls, *P* = 0.040), and normalized grey matter volume (−11.6%, *P* = 0.001), but not normalized white matter volume (*P* = 0.735) were lower in Alzheimer’s disease cases compared to controls. As per definition, Alzheimer’s disease cases had higher Braak NFT stage (*P* < 0.001), Thal phase (*P* < 0.001) and ABC score (*P* < 0.001) than controls. Regarding Alzheimer’s disease phenotypes, typical and atypical phenotypes did not differ in any demographical, radiological and pathological data. APOE genotype did not differ between phenotypes (*P* = 0.315).

**Table 1:** Clinical, radiological and pathological characteristics of included donors ABC score^31^*N*

	Control	AD	Typical AD	Atypical AD
**Clinical characteristics**				
*N* (phenotype)	10	19	10	9 (6 B/D, 3 PCA)
Gender M/F (%M)	4/6 (40%)	16/3 (84%)^*^	9/1 (90%)	7/2 (78%)
APOE genotype *N*	9	19	10	9
ε4 non-carrier	5 (56%)	7 (37%)	5 (50%)	2 (22%)
ε4 heterozygous	4 (44%)	10 (53%)	4 (40%)	6 (67%)
ε4 homozygous	–	2 (10%)	1 (10%)	1 (11%)
Age at disease onset		60 ± 10	61 ± 8	59 ± 13
years, mean ± SD		1 n.a.		1 n.a.
EAOD/LOAD (%EOAD)		14/4 (78%)	7/3 (70%)	7/1 (88%)
		1 n.a.		1 n.a.
Age at death	69 ± 7	67 ± 12	70 ± 11	64 ± 12
years, mean ± SD				
Disease duration	–	8 ± 5	10 ± 6	6 ± 3
years, mean ± SD				
Post-mortem delay	549 ± 114	478 ± 116	520 ± 95	432 ± 124
min, mean ± SD				
**Radiologic characteristics**				
NBV (L) mean ± SD	1.49 ± 0.06	1.41 ± 0.13^*^	1.42 ± 0.13	1.40 ± 0.14
NGMV (L) mean ± SD	0.76 ± 0.04	0.67 ± 0.09^**^	0.68 ± 0.07	0.67 ± 0.11^*^
NWMV (L) mean ± SD	0.72 ± 0.03	0.73 ± 0.08	0.73 ± 0.09	0.73 ± 0.08
**Pathological characteristics**				
	10	19	10	9
A 0/1/2/3	3/6/1/0	0/0/0/19^***^	0/0/0/10^***^	0/0/0/9^***^
B 0/1/2/3	3/7/0/0	0/0/4/15^***^	0/0/3/7^***^	0/0/1/8^***^
C 0/1/2/3	10/0/0/0	0/0/4/15^***^	0/0/3/7^***^	0/0/1/8^***^
Thal phase^4^*N*	10	19^***^	10^***^	9^**^
0/1/2/3/4/5	3/3/3/1/0/0	0/0/0/1/1/17	0/0/0/1/0/9	0/0/0/0/1/8
Braak NFT stage^5^*N*	10	19^***^	10^***^	9^**^
0/1/2/3/4/5/6	3/6/1/0/0/0/0	0/0/0/0/4/8/7	0/0/0/0/3/3/4	0/0/0/0/1/5/3
CAA type *N*	10	19	10	9
Type 1/type 2 (% type 1)	0/0	15/3 (83%)	7/2 (78%)	8/1 (89%)

AD, Alzheimer’s disease; B/D, behavioral/dysexecutive variant; CAA, cerebral amyloid angiopathy; EOAD, early onset Alzheimer’s disease; L, litre; LOAD, late onset Alzheimer’s disease; M/F, males/females ratio; *N*, sample size; n.a., not available; NBV, normalized brain volume; NGMV, normalized grey matter volume; NWMV, normalized white matter volume; NFT, neurofibrillary tangles; PCA, posterior cortical atrophy; SD, standard deviation.

* *P* < 0.05,

** *P* < 0.01,

*** *P* < 0.001 when compared to controls.

### MRI cortical atrophy of the temporo-parietal region in Alzheimer’s disease

**Figure 2:**
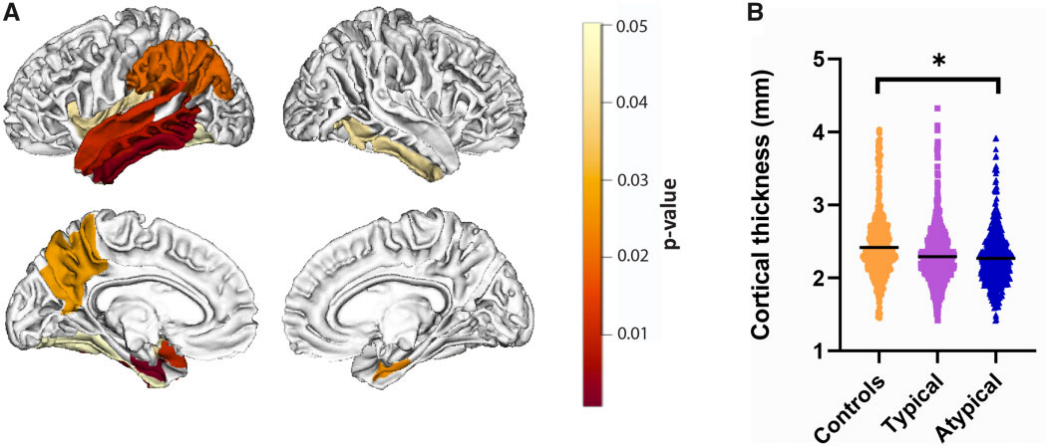
**MRI cortical atrophy in Alzheimer’s disease.** Figure (**A**) shows the atrophic patterns in Alzheimer’s disease compared to controls (when typical and atypical phenotypes were combined) across the whole cortex. The scale bar represents the false discovery rate corrected *P*-values. No significant differences in atrophic patterns were found between typical and atypical phenotypes. Graph **(B)** shows differences in global cortical thickness in controls, typical and atypical phenotypes. The boxplot represents the median, the upper and lower quartile, and the minimum and maximum values. *P < 0.05 when compared to controls. For detailed information, see Supplementary Table 5.

Alzheimer’s disease donors had significantly more cortical atrophy compared to controls (−5.7%, *P* = 0.011, see Supplementary Table 5), specifically in the inferior (*q* = 0.004), middle (*q* = 0.008) and superior temporal gyrus (*q* = 0.012), entorhinal cortex (*q* = 0.001), fusiform gyrus (*q* = 0.050), inferior parietal gyrus (*q* = 0.020), insular cortex (*q* = 0.043), supramarginal cortex (*q* = 0.020) and precuneus (*q* = 0.028) in the left hemisphere, and in the entorhinal cortex (*q* = 0.025) and inferior temporal gyrus (*q* = 0.042) in the right hemisphere (Fig. 2A). Compared to controls, atypical Alzheimer’s disease cases showed significant global cortical atrophy (−6.2%, *P* = 0.039), while typical Alzheimer’s disease cases did not (*P* = 0.116) (Fig. 2B). No significant difference in atrophy was found between Alzheimer’s disease phenotypes (*P* = 1.000) (regional uncorrected *P*-values are found in Supplementary Table 5 and visualized in Supplementary Fig. 1).

### Load and distribution of pathological hallmarks

**Figure 3:**
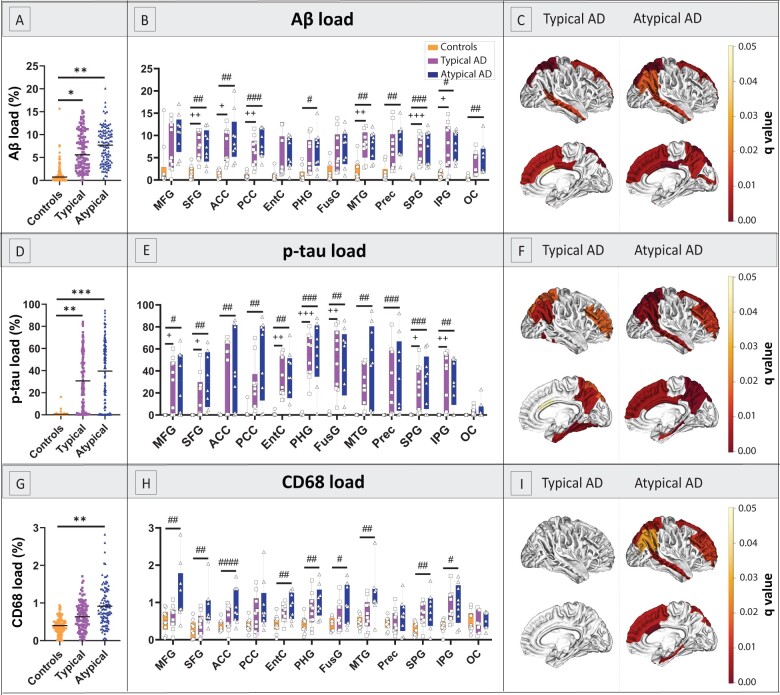
**Load and distribution of pathological hallmarks in Alzheimer’s disease phenotypes and controls.** (**A–C**) represent the load of Aβ, (**D–F**) of p-tau, and (**G–I**) of reactive microglia in the right hemisphere. The first column **(A, D and G)** shows group differences in overall pathological load with boxplots showing median, upper and lower quartile, and minimum and maximum values for each group; the middle column (**B, E and H**) shows group differences across regions; the last column (**C, F and I)** visually shows the *q*-values on the cortical surface in typical and atypical phenotypes, i.e. the same data graphically showed in the middle column. In short, we found significant differences in Aβ and p-tau distribution patterns compared to controls, but not between Alzheimer’s disease phenotypes. Additionally, atypical donors, but not typical donors, had an overall higher reactive microglia load compared to controls. ACC, anterior cingulate cortex; AD, Alzheimer’s disease; EntC, entorhinal cortex; FusG, fusiform gyrus; IPG, inferior parietal gyrus; MFG, middle frontal gyrus; MTG, middle temporal gyrus; OC, occipital cortex; PCC, posterior cingulate cortex; PHG, parahippocampal gyrus; Prec, precuneus; SFG, superior frontal gyrus; SPG, superior parietal gyrus. In the first column: *P < 0.05, **P < 0.010, ***P < 0.001 when compared to controls. In the middle column, *P*-values corrected with false discovery rate: ^+^*q* < 0.05, ^++^*q* < 0.010, ^+++^*q* < 0.001 typical Alzheimer’s disease compared to controls; ^#^*q* < 0.05, ^###^*q* < 0.010, ^###^*q* < 0.001 atypical Alzheimer’s disease compared to controls.

As expected, Alzheimer’s disease cases had significantly higher Aβ (*P* < 0.001) and p-tau load (*P* < 0.001) compared to controls (see Supplementary Table 6). By subgroup, both typical and atypical Alzheimer’s disease had higher Aβ (*P* = 0.012 and *P* = 0.001, respectively; Fig. 3A–C) and p-tau load (*P* = 0.002 and *P* < 0.001, respectively; Fig. 3D–F) compared to controls, whereas typical and atypical Alzheimer’s disease did not differ in load or regional distribution of Aβ (*P* = 1.000) or p-tau (*P* = 1.000).

Alzheimer’s disease cases had a significantly higher reactive microglia load compared to controls (*P* = 0.002, see Supplementary Table 6). By subgroup, atypical Alzheimer’s disease cases had a higher reactive microglia load than controls (*P* = 0.001), whereas typical Alzheimer’s disease cases did not (*P* = 0.104; Fig. 3G–I), suggesting that the significant difference in reactive microglia load between Alzheimer’s disease and controls was driven by the atypical Alzheimer’s disease group. No significant difference was found between Alzheimer’s disease phenotypes (*P* = 0.199), however, while typical cases did not show any regional difference in reactive microglia load compared to controls, almost all the areas examined in atypical cases showed significantly higher loads compared to controls (0.001 < *q* < 0.028, Fig. 3I), suggesting that that reactive microglia load was consistently increased across the cortical surface in the atypical phenotype, but not in the typical phenotype. For an overview of the correlations between pathological markers, see Supplementary Table 7.

### Cortical thickness associates with Thal and Braak staging

In the whole cohort, the average whole-brain cortical thickness correlated negatively with both Thal phase (*r_s_=* −0.39, *R*^2^ = 15%, *P* = 0.037) and Braak NFT stage (*r_s_* = −0.40, *R*^2^ = 16%, *P* = 0.030, Supplementary Fig. 2), suggesting an increase in cortical atrophy with disease progression.

### Aβ load weakly correlates with less cortical atrophy globally

**Figure 4:**
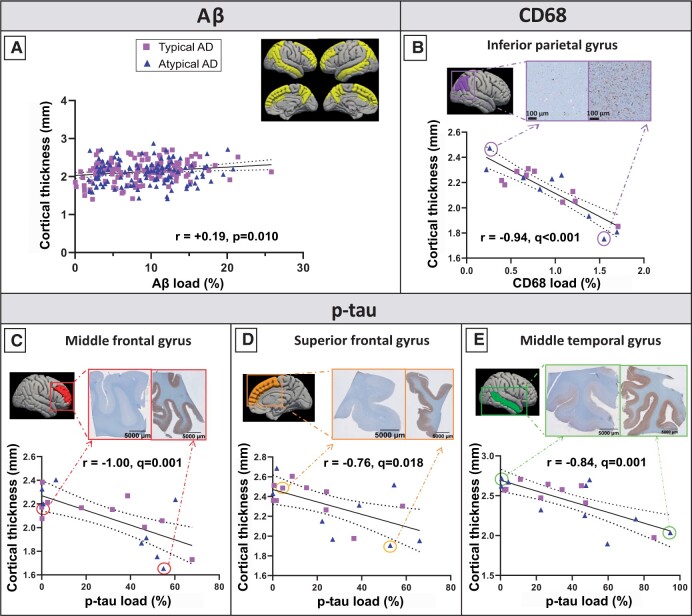
**MRI-pathology associations in Alzheimer’s disease.** (**A**) Weak positive correlation between Aβ load and cortical thickness in the Alzheimer’s disease group across regions (shown in yellow). (**B**) Strong negative correlation between reactive microglia load and cortical thickness in the inferior parietal gyrus. (**C**) Strong negative correlations between p-tau load and cortical thickness in the middle frontal gyrus, (**D**) superior frontal gyrus and (**E**) middle temporal gyrus. On the top right of each graph, two tissue sections representing a low (left) and high (right) pathological load are shown. Purple squares represent typical Alzheimer’s disease cases, and blue triangles atypical cases. A fit-line with 95% confidence interval (dashed lines) is shown for each correlation. AD, Alzheimer’s disease.

A weak positive correlation between Aβ load and cortical thickness was found in the Alzheimer’s disease group across regions (*r=* 0.19, *R*^2^*=* 3%, *P* = 0.010; Fig. 4A, Supplementary Table 8) but not in controls (*P* = 0.165). Such association survived even when adding p-tau load in the model (*r* = 0.21, *R*^2^ = 4%, *P=* 0.008). A similar association was found in typical Alzheimer’s disease (*r* = 0.22, *R*^2^ = 5%, *P* = 0.022, with p-tau as covariate: *r* = 0.23, *R*^2^ = 6%, *P=* 0.031), but not in atypical Alzheimer’s disease (*P* = 0.200). We found no associations within brain areas for any group. Overall, Aβ load weakly correlated with a slightly increased cortical thickness (relative to the atrophied cortex in Alzheimer’s disease cases compared to controls).

### P-tau load strongly correlates with regional cortical atrophy in frontal and temporal regions

No associations were found between p-tau load and cortical thickness in Alzheimer’s disease (*P* = 0.477), Alzheimer’s disease phenotypes (*P* = 0.144 for typical, and *P* = 0.856 for atypical), or controls (*P* = 0.755) across regions. However, within regions, we observed strong significant correlations in the middle (*r=−*1.00, *R*^2^ = 100%, *q* = 0.001) and superior frontal gyrus (*r = −*0.76, *R*^2^*=* 58%, *q* = 0.018), and the middle temporal gyrus (*r=−*0.84, *R*^2^*=* 71%, *q=* 0.001) in the Alzheimer’s disease group only (Fig. 4C–E, Supplementary Table 9). Overall, p-tau load strongly correlated with cortical atrophy in frontal and temporal regions in Alzheimer’s disease.

### Reactive microglia load correlates with cortical atrophy in the parietal region

No associations were found between reactive microglia load and cortical thickness in the Alzheimer’s disease group (*P* = 0.487) nor controls (*P* = 0.242) across regions. Similarly, we found no associations in typical (*P* = 0.747) nor atypical (*P* = 0.285) Alzheimer’s disease phenotypes. However, within regions, we found a strong, negative significant association in the right inferior parietal gyrus in Alzheimer’s disease donors (*r =* −0.94, *R*^2^ = 89%, *q<* 0.001) (Fig. 4B), which survived also when p-tau load was included in the model as covariate (*r=−*0.86, *R*^2^*=* 74%, *q<* 0.001). Overall, reactive microglia load strongly correlated with cortical atrophy in the inferior parietal gyrus in Alzheimer’s disease.

### Combined contribution of pathological hallmarks to cortical thickness

A regression model was run to investigate the combined contribution of Aβ, p-tau and reactive microglia load on cortical thickness of each brain area, and to investigate which had the strongest association with cortical atrophy. While we found no associations in controls, we found significant associations in Alzheimer’s disease in the middle frontal gyrus (*r* = 0.88, *R*^2^ = 77%, *q=* 0.031), and the inferior parietal gyrus (*r=* 0.92, *R*^2^ = 84%, *q<* 0.001), explaining up to 84% of the variance in cortical thickness. In Alzheimer’s disease, p-tau load was the major contributor in the middle frontal gyrus (*q* = 0.007), while reactive microglia load was the main contributor in the inferior parietal gyrus (*q* = 0.001). When the Alzheimer’s disease group was split up in typical and atypical Alzheimer’s disease, no areas showed significant regression models.

### From post-mortem *in situ* to ante-mortem *in vivo*

Additionally, we investigated the association between ante-mortem *invivo* and post-mortem *insitu* MRI scans within the same Alzheimer’s disease donors. Cortical thickness assessment of scans acquired <1 year before death tended to have a stronger correlation with post-mortem cortical thickness than ante-mortem scans acquired 8–10 years before death (*r* ranged from *0.98* in ante-mortem scan with a 2-year interval from death to *0.69* in ante-mortem scan with a 10-year interval from death, *p* < 0.001 for all, Supplementary Table 10). Moreover, we investigated the correlation between p-tau load and both ante-mortem and post-mortem cortical thickness in the three brain areas that showed significant correlations in our study (see Paragraph 3.5). Cortical thickness measured from ante-mortem scans acquired shortly before death (<1 year, *n = 3*) showed high concordance with post-mortem cortical thickness and its relationship with histopathology, while cortical thickness measured from ante-mortem scans acquired 8–10 years before death (*n* = 2) showed more discordance with post-mortem cortical thickness, especially in the middle temporal gyrus (Supplementary Fig. 3A and B). In one Alzheimer’s disease case who had three ante-mortem scans in addition to the post-mortem scan, we found a progressively increased pattern of discordance with more years between ante-mortem scan and death, with an mean deviation from post-mortem cortical thickness of 22 μm ± 17 at 1-month, 24 μm ± 17 at 2-year, and 107 μm ± 37 at 3-year intervals (Supplementary Fig. 3C).

## Discussion

Using a combined post-mortem *insitu* MRI and histopathology approach, we investigated the associations between MRI cortical thickness and Aβ, p-tau and reactive microglia load in clinically-defined and pathologically-confirmed Alzheimer’s disease and control donors, and explored these associations in typical and atypical Alzheimer’s disease phenotypes. To do this, we correlated cortical thickness with pathological load both globally (all regions together) and regionally (within specific regions). Associations between the histopathological hallmarks and MRI cortical atrophy were found in the Alzheimer’s disease group, and not in controls. In Alzheimer’s disease, Aβ and p-tau load contributed differently to cortical thickness: Aβ associated weakly and globally with reduced cortical atrophy (taking all cortical regions together), while p-tau accumulation strongly associated to cortical atrophy in temporal and frontal regions. In the cortex of Alzheimer’s disease donors, the strongest contributors to cortical atrophy were p-tau load in the frontal and temporal cortices, and reactive microglia load in the parietal region.

On MRI, our Alzheimer’s disease cohort showed pronounced cortical atrophy in the temporoparietal region of the left hemisphere, consistent with the classic Alzheimer’s disease signature and independent of the phenotypical presentation.^2^^,^^3^^,^^9^

We found a higher Aβ load in both Alzheimer’s disease phenotypes compared to controls, but we did not observe a difference in Aβ load nor distribution between Alzheimer’s disease phenotypes, which is in line with previous studies.^8^^,^^9^ Furthermore, we found a high p-tau load in Alzheimer’s disease excluding the occipital cortex, consistent with the fact that this region is the last affected according to Braak NFT staging^5^ and 12 out of 19 of our cases not having reached this stage yet. We did not find any significant differences in p-tau load nor distribution in clinically-defined Alzheimer’s disease phenotypes, although a difference in distribution has been described previously.^8^^,^^33–35^ However, most of those studies used pathologically-defined Alzheimer’s disease donors as opposite to our clinically defined cohort,^8^^,^^34^^,^^35^ and the only study that used clinically defined phenotypes did not find any significant difference in NFT load or distribution in the behavioural/dysexecutive variant compared to typical Alzheimer’s disease cases,^33^ which represents 6 out of 9 cases of our atypical Alzheimer’s disease cohort.

In line with the literature,^6^^,^^7^ we show that Alzheimer’s disease cases had a higher reactive microglia load compared to controls, which was driven by the atypical Alzheimer’s disease group in our cohort. Atypical Alzheimer’s disease cases had a higher reactive microglia load compared controls, while typical Alzheimer’s disease cases did not. Neuroinflammation tends to be stronger in relatively young Alzheimer’s disease patients compared to the oldest patients,^36^ however, our atypical cohort did not have a younger age compared to our typical Alzheimer’s disease group. As such, the underlying disease mechanisms that lead to an increased inflammatory response remains to be elucidated.

**Figure 5:**
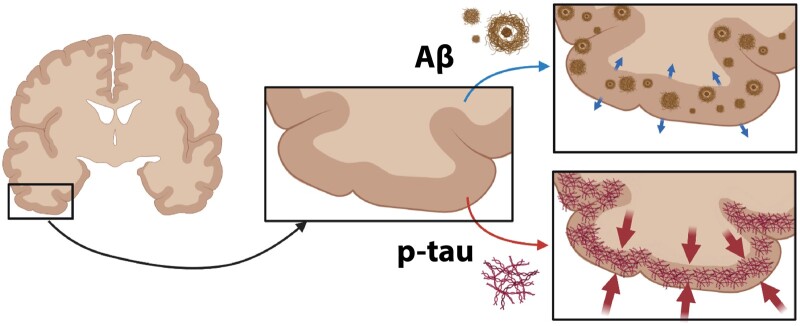
**Summary figure: differential effects of p-tau and Aβ load on cortical thickness in Alzheimer’s disease.** Aβ weakly correlates with a reduced cortical atrophy (top; small blue arrows), while p-tau load strongly correlates with cortical atrophy (bottom; big red arrows) in Alzheimer’s disease.

When associating our MRI findings with Alzheimer’s disease pathological hallmarks, we found a weak positive association between Aβ load and cortical thickness in Alzheimer’s disease, suggesting that a higher Aβ load associated to a slightly increased cortical thickness relatively to the atrophic Alzheimer’s cortex (Fig. 5). Previous studies also reported that Aβ deposition is widespread across the cortex in Alzheimer’s disease, however, they were not consistent in the association with cortical atrophy nor in the direction of the association; some PET studies showed no correlation between Aβ and cortical thickness or grey matter volume,^9^^,^^11^^,^^12^^,^^37^ while other CSF studies found a positive correlation.^38–42^ The latter argued the existence of a “two-phase phenomenon” along the Alzheimer’s disease continuum, according to which cortical thickness follows a biphasic trajectory: in preclinical phases there is a cortical thickening, suggesting a relationship with amyloid deposition, and in the late clinical phases cortical atrophy occurs, indicating the (additional) influence of p-tau accumulation.^38–43^ Our results being similar to these latter CSF studies is not due to the inclusion of preclinical Alzheimer’s disease cases, since the positive association was found in our clinical Alzheimer’s disease group, but more likely due to the use of immunohistochemical quantification. In fact, immunohistochemistry is sensitive to pick up even small diffuse accumulations of Aβ (which is limited with PET imaging).^44–46^ Therefore, it is possible that our study revealed a positive association between Aβ and cortical thickness in late clinical stages similarly to preclinical Alzheimer’s disease.

We found that the regional increase in p-tau load strongly associated with cortical atrophy in frontal and temporal regions in Alzheimer’s disease (Fig. 5). These results are in line with several PET studies which reported regional correlations between tau PET and cortical thickness or grey matter volume.^9–13^^,^^37^ Even if the exact mechanisms of p-tau-mediated neurodegeneration are still unclear, p-tau has been shown to be closely related to axonal transports deficits and neuronal and synaptic loss, leading to volume loss, hence cortical atrophy.^47^ A striking finding in our study is the variance in cortical thickness explained by p-tau load, which ranges between 58% in the superior frontal gyrus to 100% in the middle frontal gyrus, suggesting that p-tau load is indeed one of the main contributors to cortical atrophy in Alzheimer’s disease. While temporal regions are expected to be hit by p-tau-associated atrophy,^3^ our results indicate that frontal regions are also particularly vulnerable to p-tau pathology, which might be due to our inclusion of atypical cases of the behavioural/dysexecutive phenotype.

Regarding the association between cortical thickness and neuroinflammation, we found a strong association between reactive microglia load and cortical atrophy in the parietal region in Alzheimer’s disease. Similar findings have been reported in a PET study, where the PET marker ^11^CPK11195 for microglial activation correlated with parieto-occipital thinning in Alzheimer’s disease, including the inferior parietal gyrus.^16^ The temporoparietal region reveals neuroinflammation in Alzheimer’s disease,^48^ which can contribute to structural damage. Since the temporal region is one of the first to be affected,^5^ any correlational analysis with structural imaging is likely to suffer from a floor effect in end-stage Alzheimer’s disease cases.^16^ Therefore, cortical thinning is, most likely, significantly correlated with neuroinflammation only in parietal areas. When chronically activated, microglia tend to transform to a dystrophic, senescent phenotype, which brings them to lose their neuroprotective functions and to become detrimental and neurotoxic, thus accelerating the disease course.^7^ Senescent reactive microglia are known to release cytokines, reactive oxygen species and pro-inflammatory factors,^6^ which can contribute to synaptic and neuronal loss,^7^ hence neurodegeneration.

To further validate our results, we explored the correlation between post-mortem and ante-mortem cortical thickness extrapolated from scans acquired shortly before death and several years prior to it, and its correlation to histopathology. Our findings show that post-mortem *insitu* scans showed high concordance with ante-mortem *invivo* scans acquired few months prior to death. On the other hand, when the ante-mortem scans were acquired several years before death, the associations showed discrepancies. To conclude, this confirms that post-mortem MRI can be used as a proxy for *invivo* MRI.^49^

The main strength of this study is that MRI and gold-standard immunohistological data were collected from the same donor at the same moment in time. All donors had pathological confirmation of clinical diagnosis, as clinical–pathological discrepancies occur in 10% of cases,^50^ and may obscure *invivo* studies. In addition, comprehensive clinical and pathological datasets were available, and both ante-mortem and post-mortem MRI were collected for a subset of patients, making this study encompassing clinical, pathological and radiological data. However, there are also limitations, such as the small group sample sizes, the heterogeneity of our atypical Alzheimer’s disease cohort, and the fact that the ante-mortem MRI scans have not been collected systematically, having therefore slightly different parameters. It is noteworthy to mention that 78% of our Alzheimer’s disease cohort had an early disease onset (EOAD). While this is common in the atypical phenotype, it is not in the typical phenotype.^1^ As cortical atrophy^51^ and neuropathological load^52^ are usually more abundant in EOAD than late onset Alzheimer’s disease (LOAD), the fact that our typical Alzheimer’s cohort was mostly composed of EOAD cases (70%) might have obscured possible differences in cortical atrophy and neuropathological load in the comparison between phenotypes. At the same time, our typical and atypical Alzheimer’s disease cohorts were comparable in age, which was particularly useful when comparing inflammation (i.e. reactive microglia load).^36^ Moreover, our Alzheimer’s cohort was mostly composed of males (16 males and 3 females). While young atypical cases are more often males,^1^ the fact that our early onset typical Alzheimer’s disease cohort was mostly composed of males was purely driven by chance, but made our typical and atypical cohorts gender-matched. Additionally, measures of pathology are expressed as percentage of staining in a region of interest, measure that inherently includes a measure of atrophy, introducing a bias when correlating this measure with MRI cortical atrophy. However, quantitative measures such as percentage area load are more representative and less biased than semi-quantitative measures, such as scoring. Lastly, since we used only one antibody for p-tau, glial and neuronal tau could not be differentiated, and similarly, different Aβ variants were not differentiated. Also, we investigated three markers that might contribute to cortical thickness changes, while it is likely that also other cellular and molecular components contribute to cortical atrophy, such as synaptic and axonal degeneration. Future research should therefore investigate the MRI-pathology associations in a larger cohort and include more neurodegenerative markers and molecular profiling to further validate MRI atrophy patterns in Alzheimer’s disease.

Taken together, our findings show that, in Alzheimer’s disease, Aβ load correlates with a slightly increased cortical thickness relatively to the atrophic Alzheimer’s disease cortex, while p-tau load is the strongest contributor to regional cortical atrophy in frontal and temporal regions, and reactive microglia load is the strongest correlate of cortical atrophy in the parietal region. An exploration within Alzheimer’s disease phenotypes showed an increased reactive microglia load in atypical Alzheimer’s disease, but not typical Alzheimer’s disease, compared to controls, even though no or only subtle differences were found in MRI-pathology associations, indicating that the histopathological correlates of cortical atrophy might be similar between Alzheimer’s disease phenotypes included in our study. Moreover, we show that post-mortem *insitu* MRI can be used as proxy for ante-mortem *invivo* MRI. In conclusion, our results show that distinct histopathological markers correlate differently with cortical atrophy, highlighting their different roles in the neurodegenerative process, therefore contributing to the understanding of the pathological underpinnings of MRI atrophic patterns in Alzheimer’s disease.

## Supplementary material

Supplementary material is available at *Brain Communications* online.

## Supplementary Material

fcab281_Supplementary_Data

## Abbreviations

Aβ =: amyloid-beta
EOAD =: early disease onset
FDR =: false discovery rate
LOAD =: late disease onset
NFT =: neurofibrillary tangles
p-tau =: phosphorylated-tau
