# Supplementary material for: Amyloid-β, p-tau and reactive microglia are pathological correlates of MRI cortical atrophy in Alzheimer’s disease
Source: Brain Commun. 2021 Nov 24;3(4):fcab281. doi: 10.1093/braincomms/fcab281 (PMC8677327; doi:10.1093/braincomms/fcab281)
Supplement: fcab281_Supplementary_Data [file fcab281_supplementary_data.pdf]

Supplementary tables

Supplementary Table 1. Donor characteristics

Case number	Phenotype	Gender	Age at death (years)	Disease duration (years)	Early and late onset	PMD (minutes)	Cause of death	NBV (L)	NGMV (L)	ABC score[32]	Thal phase[4]	Braak NFT stage[5]
**Controls**												
**1**	/	M	68	-		510	Euthanasia	1.61	0.85	A1 B1 C0	2	1
**2**	/	F	63	-		490	Euthanasia	1.53	0.75	A0 B0 C0	0	0
**3**	/	F	72	-		440	Hart failure	1.52	0.80	A0 B0 C0	0	0
**4**	/	F	69	-		765	Pulmonary embolism	1.38	0.70	A1 B1 C0	1	1
**5**	/	M	59	-		480	Euthanasia	1.49	0.77	A1 B1 C0	2	1
**6**	/	M	77	-		685	Pneumonia	1.46	0.72	A1 B1 C0	1	1
**7**	/	F	78	-		600	Unknown	1.52	0.78	A1 B1 C0	1	1
**8**	/	F	59	-		490	Euthanasia	1.46	0.78	A0 B0 C0	0	0
**9**	/	F	71	-		410	Lung Carcinoma	1.48	0.77	A1 B1 C0	2	1
**10**	/	M	74	-		620	Euthanasia	1.43	0.72	A2 B1 C0	3	2
**Typical AD**												
**11**	Typical	M	60	2	EOAD	515	Euthanasia	1.55	0.76	A3 B3 C3	5	6
**12**	Typical	M	68	6	EOAD	555	Euthanasia	1.43	0.72	A3 B3 C3	5	5
**13**	Typical	M	69	11	EOAD	715	Pulmonary infection	1.66	0.69	A3 B3 C3	5	5
**14**	Typical	M	84	13	LOAD	353	Euthanasia	1.43	0.72	A3 B2 C2	5	4
**15**	Typical	F	80	7	EOAD	425	Euthanasia	1.44	0.71	A3 B2 C2	5	4
**16**	Typical	M	53	5	EOAD	540	Palliative sedation	1.26	0.60	A3 B3 C3	5	6
**17**	Typical	M	64	12	EOAD	475	Dehydration	1.25	0.57	A3 B3 C3	5	6
**18**	Typical	M	84	23	EOAD	515	Euthanasia	1.25	0.61	A2 B2 C2	3	4
**19**	Typical	M	77	9	LOAD	545	Excess of pills	1.40	0.67	A3 B3 C3	5	6
**20**	Typical	M	65	7	LOAD	560	Euthanasia	1.46	0.77	A3 B3 C3	5	5

**Atypical AD**												
**21**	PCA	M	65	7	EOAD	470	Cardiac arrest	1.51	0.77	A3 B3 C3	4	5
**22**	B/D	M	59	n.a.	n.a.	210	Euthanasia	1.58	0.80	A3 B3 C3	5	5
**23**	B/D	F	78	4	LOAD	450	Dehydration	1.36	0.66	A3 B3 C3	5	5
**24**	PCA	M	62	8	EOAD	495	Palliative sedation	1.16	0.53	A3 B3 C3	5	6
**25**	B/D	M	37	5	EOAD	671	Euthanasia	1.51	0.73	A3 B3 C3	5	6
**26**	PCA	M	67	9	EOAD	395	Dehydration	1.25	0.58	A3 B3 C3	5	6
**27**	B/D	M	77	4	EOAD	420	Euthanasia	1.54	0.77	A3 B2 C2	5	4
**28**	B/D	F	59	3	EOAD	335	Swallowing disorder	1.32	0.65	A3 B3 C3	5	5
**29**	B/D	M	73	10	EOAD	440	Cachexia	1.37	0.49	A3 B3 C3	5	5

Abbreviations: AD = Alzheimer's disease; PCA = posterior cortical atrophy; B/D = behavioral / dysexecutive variant; M = male; F = female; n.a. = not available; EOAD = early onset Alzheimer's disease; LOAD = late onset Alzheimer's disease; PMD = post-mortem delay; NBV = normalized brain volume; L = liter; NGMV = normalized grey matter volume.

Supplementary Table 2. Information on in-vivo MRI scans.

Case number	Scanner	Dimensions	In-plane resolution	TE (ms)	TR (ms)	Interval (years)
**12**	3T GE MR750	176x256x256	$1mm^3$	3	7.8	2
**12**	3T GE MR750	176x256x256	$1mm^3$	3	7.8	5
**14**	unknown	176x256x256	$1mm^3$	3.4	unknown	2
**14**	3T GE MR750	176x256x256	$1mm^3$	3	7.8	7
**16**	3T GE MR750	176x256x256	$1mm^3$	3.2	7.8	4
**17**	unknown	192x150x256	$1mm^3$	5.2	unknown	10
**18**	3T GE MR750	288x288x180	$1mm^3$	3	7.8	6
**18**	3T GE MR750	176x256x256	$1mm^3$	3	7.8	7
**19**	Philips 3T Achieva	256x256x192	$1mm^3$	4.5	7.9	2
**19**	Philips 3T Achieva	256x256x192	$1mm^3$	4.5	7.9	4
**20**	Philips 3T Achieva	256x256x192	$1mm^3$	4.5	7.9	<1
**20**	Philips 3T Achieva	256x256x192	$1mm^3$	4.5	7.9	2
**20**	3T GE MR750	176x256x256	$1mm^3$	3.2	7.8	3
**21**	Philips 3T Achieva	256x256x192	$1mm^3$	4.5	7.9	1
**24**	3T GE MR750	176x256x256	$1mm^3$	3	7.8	5
**25**	3T GE MR750	174x512x512	$1mm^3$	2.9	7.8	<1
**26**	3T GE MR750	176x256x256	$1mm^3$	3.2	7.8	5
**27**	Philips 3T Achieva	256x256x192	$1mm^3$	4.5	7.9	<1
**28**	3T GE MR750	176x256x256	$1mm^3$	3.2	7.8	2
**29**	3T GE MR750	176x256x256	$1mm^3$	3.2	7.8	4

The table shows the information about the ante-mortem *in-vivo* 3T MRI of 14 out of 19 Alzheimer's disease cases included in our post-mortem cohort. In addition, 5 out of 14 cases had more than one ante-mortem MRI scan at different intervals from death, measured in years. Case numbers match those in Supplementary Table 1. Legend: TE = time to echo, TR = repetition time; ms = milliseconds.

Supplementary Table 3. Brain areas included in the study.

	**Right hemisphere**	**Left hemisphere**
**Groups**	Controls (n=10), typical (n=10) and atypical AD (n=9)	Typical (n=7) and atypical AD (n=6)
**Brain Areas**		
**Middle frontal gyrus**	*x*	*x*
**Superior frontal gyrus**	*x*	*x*
**Anterior cingulate cortex**	*x*	*x*
**Posterior cingulate cortex**	*x*	
**Superior temporal gyrus**		*x*
**Middle temporal gyrus**	*x*	*x*
**Entorhinal cortex**	*x*	
**Parahippocampal gyrus**	*x*	
**Fusiform gyrus**	*x*	
**Precuneus**	*x*	*x*
**Superior parietal gyrus**	*x*	*x*
**Inferior parietal gyrus**	*x*	*x*
**Occipital cortex (V1)**	*x*	*x*
	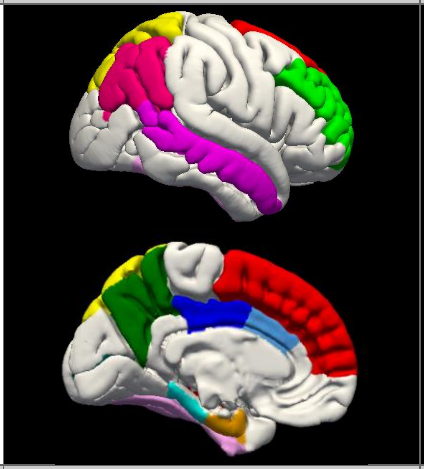	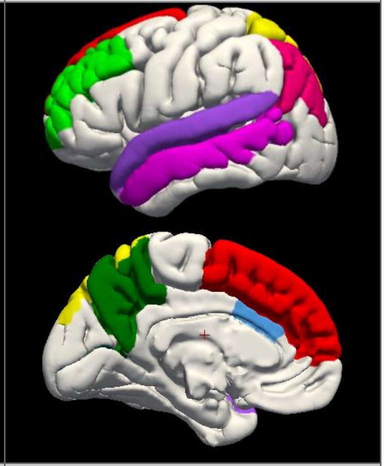
**Total**	*=12*	*=9*

Abbreviations: AD = Alzheimer's disease

Supplementary Table 4. Information on primary antibodies.

Antibody	Antigen	Species	Origin details	Dilution	Incubation time	Antigen retrieval	Detection method
**Aβ, clone 4G8**	Aβ amino acid sequence 17-24	Mouse igG2b	BioLegend, San Diego, USA	1:8000	4°C overnight	Autoclave Citrate buffer (pH 6.0, 10 minutes)	EnVision
**p-tau, clone AT8**	Tau phosphorylated at Ser202 and Thr205	Mouse igG1	ThermoFisher, Pittsburgh, USA	1:800	4°C overnight	Autoclave Citrate buffer (pH 6.0, 10 minutes)	EnVision
**CD68, clone KP1**	CD68	Mouse igG1	Dako, Glostrup, Denmark	1:1200	4°C overnight	Autoclave Citrate buffer (pH 6.0, 10 minutes)	EnVision

Supplementary Table 5. Mean and standard deviation of MRI cortical thickness per brain area in each group.

		Controls (n=10)	AD (n=19)	Typical AD (n=10)	Atypical AD (n=9)	Q-value
ctx-lh-bankssts	1	2,26±0,09	2,07±0,23 °	2,06±0,24	2,08±0,24	°
ctx-lh-caudalanteriorcingulate	2	2,33±0,24	2,31±0,32	2,20±0,28	2,42±0,33	ns
ctx-lh-caudalmiddlefrontal	3	2,34±0,11	2,22±0,22 °	2,22±0,17	2,22±0,27	°
ctx-lh-cuneus	4	1,88±0,11	1,86±0,15	1,84±0,11	1,87±0,18	ns
ctx-lh-entorhinal	5	3,48±0,22	2,81±0,50 **	2,75±0,53 °	2,87±0,49 °	q=0.001
ctx-lh-fusiform	6	2,63±0,13	2,40±0,22 *	2,43±0,24	2,37±0,22 °	q=0.050
ctx-lh-inferiorparietal	7	2,30±0,13	2,11±0,15 *	2,08±0,17 °	2,13±0,12	q=0.020
ctx-lh-inferiortemporal	8	2,74±0,14	2,50±0,19 **	2,49±0,23 °	2,50±0,16 °	q=0.004
ctx-lh-isthmuscingulate	9	2,12±0,15	2,00±0,18	1,96±0,18	2,06±0,17	ns
ctx-lh-lateraloccipital	10	2,09±0,11	2,07±0,14	2,04±0,12	2,10±0,15	ns
ctx-lh-lateralorbitofrontal	11	2,74±0,19	2,61±0,23	2,59±0,20	2,62±0,27	ns
ctx-lh-lingual	12	1,98±0,05	1,93±0,18	1,91±0,17	1,96±0,20	ns
ctx-lh-medialorbitofrontal	13	2,37±0,13	2,24±0,20 °	2,24±0,19	2,24±0,22	°
ctx-lh-middletemporal	14	2,72±0,12	2,45±0,20 **	2,42±0,19 °	2,48±0,21 °	q=0.008
ctx-lh-parahippocampal	15	2,63±0,22	2,41±0,34	2,33±0,26	2,50±0,42	ns
ctx-lh-paracentral	16	2,21±0,07	2,23±0,15	2,22±0,14	2,24±0,17	ns
ctx-lh-parsopercularis	17	2,36±0,08	2,27±0,14	2,26±0,15	2,29±0,12	ns

ctx-lh-parsorbitalis	18	2,72±0,19	2,57±0,24	2,52±0,25	2,62±0,24	ns
ctx-lh-parstriangularis	19	2,32±0,10	2,30±0,19	2,26±0,21	2,34±0,17	ns
ctx-lh-pericalcarine	20	1,74±0,16	1,68±0,15	1,67±0,17	1,68±0,14	ns
ctx-lh-postcentral	21	2,00±0,11	1,97±0,10	1,99±0,08	1,96±0,11 #	#
ctx-lh-posteriorcingulate	22	2,18±0,27	2,12±0,22	2,13±0,24	2,11±0,21	ns
ctx-lh-precentral	23	2,33±0,08	2,31±0,16	2,31±0,13	2,30±0,19	ns
ctx-lh-precuneus	24	2,25±0,14	2,07±0,14 *	2,06±0,16 °	2,08±0,12	q=0.028
ctx-lh-rostralanteriorcingulate	25	2,62±0,20	2,46±0,29	2,55±0,29	2,36±0,28	ns
ctx-lh-rostralmiddlefrontal	26	2,28±0,13	2,12±0,18	2,12±0,17	2,11±0,21	ns
ctx-lh-superiorfrontal	27	2,44±0,13	2,33±0,22 °	2,33±0,19	2,32±0,26	°
ctx-lh-superiorparietal	28	2,09±0,12	1,96±0,12 °	1,97±0,10	1,94±0,14 °	°
ctx-lh-superiortemporal	29	2,53±0,14	2,37±0,17 *	2,37±0,17 °	2,37±0,18 °	q=0.012
ctx-lh-supramarginal	30	2,34±0,14	2,13±0,15 *	2,15±0,16 °	2,12±0,15 °	q=0.020
ctx-lh-frontalpole	31	2,63±0,19	2,47±0,27 °	2,42±0,29	2,52±0,25	°
ctx-lh-temporalpole	32	3,64±0,22	3,24±0,41 °	3,24±0,47	3,25±0,37	°
ctx-lh-transversetemporal	33	2,11±0,12	2,12±0,21	2,10±0,16	2,14±0,25	ns
ctx-lh-insula	34	2,71±0,08	2,54±0,21 *	2,60±0,22	2,47±0,19 °	q=0.043
ctx-rh-bankssts	35	2,30±0,10	2,16±0,23	2,19±0,24	2,12±0,23	ns
ctx-rh-caudalanteriorcingulate	36	2,25±0,22	2,22±0,24	2,2±0,186	2,18±0,31	ns
ctx-rh-caudalmiddlefrontal	37	2,34±0,15	2,19±0,20	2,20±0,17	2,18±0,25	ns
ctx-rh-cuneus	38	1,86±0,08	1,88±0,17	1,88±0,20	1,87±0,13	ns
ctx-rh-entorhinal	39	3,67±0,25	3,09±0,60 *	3,10±0,73 °	3,08±0,45 °	q=0.025
ctx-rh-fusiform	40	2,66±0,15	2,45±0,26 °	2,48±0,25	2,41±0,29	°
ctx-rh-inferiorparietal	41	2,31±0,11	2,15±0,19 °	2,16±0,15	2,13±0,24	°
ctx-rh-inferiortemporal	42	2,79±0,15	2,54±0,24 *	2,63±0,18	2,44±0,27 °	q=0.042
ctx-rh-isthmuscingulate	43	2,10±0,24	2,03±0,24	2,11±0,19	1,94±0,27	ns
ctx-rh-lateraloccipital	44	2,15±0,08	2,14±0,16	2,15±0,15	2,13±0,17	ns
ctx-rh-lateralorbitofrontal	45	2,72±0,17	2,61±0,23	2,70±0,16	2,51±0,26	ns
ctx-rh-lingual	46	1,98±0,11	1,95±0,15	1,93±0,16	1,98±0,15	ns
ctx-rh-medialorbitofrontal	47	2,48±0,1	2,29±0,25 °	2,34±0,24	2,23±0,27 °	°
ctx-rh-middletemporal	48	2,70±0,08	2,45±0,26 °	2,52±0,21	2,37±0,30 °	°
ctx-rh-parahippocampal	49	2,69±0,35	2,40±0,36	2,34±0,39	2,46±0,33	ns
ctx-rh-paracentral	50	2,23±0,12	2,28±0,12	2,28±0,15	2,29±0,09	ns
ctx-rh-parsopercularis	51	2,32±0,14	2,24±0,20	2,29±0,15	2,19±0,24	ns
ctx-rh-parsorbitalis	52	2,63±0,11	2,51±0,24	2,55±0,21	2,47±0,27	ns
ctx-rh-parstriangularis	53	2,28±0,07	2,21±0,17	2,23±0,14	2,18±0,21	ns

ctx-rh-pericalcarine	54	1,62±0,13	1,67±0,15	1,68±0,16	1,66±0,14	ns
ctx-rh-postcentral	55	1,96±0,08	1,95±0,13	1,99±0,13	1,90±0,12 #	#
ctx-rh-posteriorcingulate	56	2,06±0,33	2,10±0,27	2,21±0,18	1,97±0,30	ns
ctx-rh-precentral	57	2,30±0,11	2,30±0,15	2,30±0,17	2,29±0,15	ns
ctx-rh-precuneus	58	2,28±0,13	2,11±0,20 °	2,12±0,17	2,10±0,23	°
ctx-rh-rostralanteriorcingulate	59	2,70±0,20	2,58±0,36	2,63±0,27	2,53±0,45	ns
ctx-rh-rostralmiddlefrontal	60	2,26±0,09	2,08±0,22 °	2,12±0,18	2,04±0,26	°
ctx-rh-superiorfrontal	61	2,46±0,13	2,33±0,24	2,38±0,18	2,27±0,29	ns
ctx-rh-superiorparietal	62	2,08±0,13	1,97±0,16	2,01±0,09	1,93±0,20	ns
ctx-rh-superiortemporal	63	2,55±0,10	2,38±0,23 °	2,43±0,20	2,33±0,27 °	°
ctx-rh-supramarginal	64	2,33±0,14	2,20±0,18	2,27±0,10	2,14±0,23	ns
ctx-rh-frontalpole	65	2,63±0,27	2,38±0,28 °	2,40±0,27	2,36±0,30	°
ctx-rh-temporalpole	66	3,80±0,16	3,34±0,50 °	3,38±0,51	3,29±0,51	°
ctx-rh-transversetemporal	67	2,18±0,14	2,16±0,29	2,13±0,11	2,20±0,19	ns
ctx-rh-insula	68	2,69±0,10	2,54±0,29 °	2,61±0,22	2,47±0,34 °	°
**Whole cortex**		**2,42 ± 0.43**	**2,28 ± 0.38***	**2,29 ± 0.38**	**2,27 ± 0.39***	

Data are presented as mean ± standard deviation. Regions selected for the study are highlighted in light grey. Legend: AD = Alzheimer’s disease; ctx = cortex; FDR = false-discovery rate; lh = left hemisphere; rh = right hemisphere. *$q<0.05$, **$q<0.01$, °did not survive the FDR correction when compared to controls, # did not survive the FDR correction when compared to typical AD, ns = not significant. For visualization of uncorrected p-values, check supplementary figure 1.

Supplementary Table 6. Mean and standard deviation of pathological hallmarks.

Pathological marker	Controls	AD	Typical AD	Atypical AD
**Aβ load (%)**	1.32 ± 2.04	7.03 ± 4.07 ***	6.45 ± 4.08 *	7.66 ± 3.99 **
**p-tau load (%)**	0.36 ± 1.66	33.81 ± 27.76 ***	28.76 ± 26.28 **	39.58 ± 28.39 ***
**CD68 load (%)**	0.40 ± 0.21	0.77 ± 0.47 **	0.64 ± 0.37	0.91 ± 0.53 **

Averaged data from brain regions of the right hemisphere. All the values are expressed in mean ± standard deviation. Legend: AD = Alzheimer’s disease. *$p<0.05$, **$p<0.01$, ***$p<0.001$ when compared to controls.

Supplementary Table 7. Associations between pathological markers in different groups.

Pathological marker	Aβ load	p-tau load	CD68 load
**Controls**			
**p-tau load**	r= -0.03, p=0.701	-	r= -0.08, p=0.428
**CD68 load**	**r=0.27, p=0.018***	r= -0.08, p=0.428	-
**AD**			
**p-tau load**	r=0.01, p=0.909	-	**r=0.32, p<0.001*** **
**CD68 load**	r=0.04, p=0.408	**r=0.32, p<0.001*** **	-
**Typical AD**			
**p-tau load**	r=0.04, p=0.660	-	**r=0.18, p=0.029***
**CD68 load**	r=0.08, p=0.888	**r=0.18, p=0.029***	-
**Atypical AD**			
**p-tau load**	r= -0.02, p=0.767	-	**r=0.40, p<0.001*** **
**CD68 load**	r=0.08, p=0.295	**r=0.40, p<0.001*** **	-

The associations between histopathological markers in controls, Alzheimer’s disease, typical and atypical phenotypes are shown in all the brain areas combined. In each cell, if the correlation was significant, the correlation coefficient (r) of the association and its the p-value are shown. Legend: AD = Alzheimer’s disease.

Supplementary Table 8. Correlations between global cortical thickness and Aβ load.

Group	r	R^2	P-value
Controls	0.11	1%	p=0.165
**AD**	**0.19**	**3%**	**p=0.010***
**Typical AD**	**0.22**	**5%**	**p=0.022***
Atypical AD	0.17	3%	p=0.200

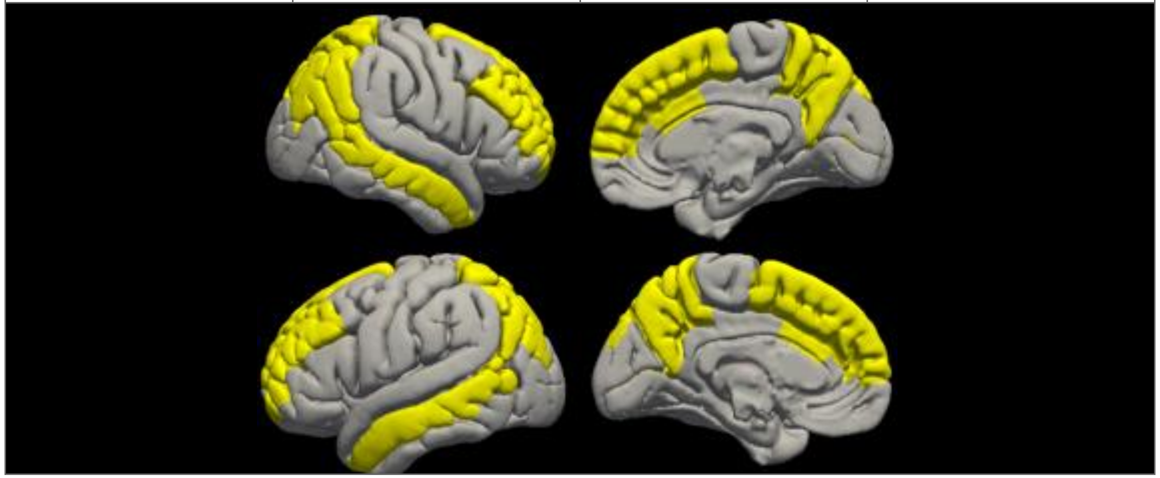

The table shows the associations of cortical thickness with Aβ load in Alzheimer's disease, controls, typical and atypical phenotypes. The columns show the correlation coefficient (r), explained variance in cortical thickness by Aβ load (R^2) and p-value of the correlations tested in the brain regions shown in the last column (in yellow), which were the same for left and right hemisphere: middle frontal and superior frontal gyrus, anterior cingulate cortex, middle temporal gyrus, superior and inferior parietal gyrus, precuneus and occipital cortex. Legend: AD = Alzheimer's disease.

Supplementary Table 9. Correlations between regional cortical thickness on MRI and p-tau load in Alzheimer's Disease.

Region	r	R^2	Q-value	Regional r values
**Middle frontal gyrus**	**-1.00**	**100%**	**q=0.001*** **	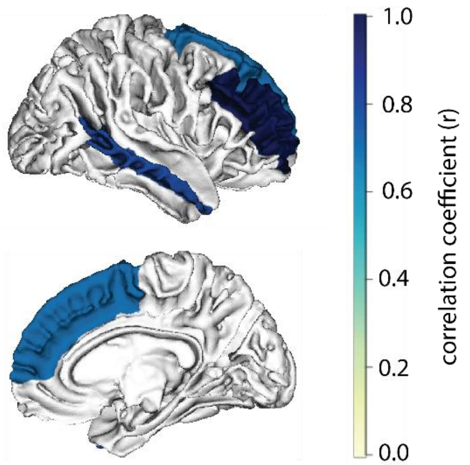
**Superior frontal gyrus**	**-0.76**	**58%**	**q=0.018***	
Anterior cingulate cortex	-0.37	24%	q=0.799	
Posterior cingulate cortex	-0.39	15%	q=0.415	
**Middle temporal gyrus**	**-0.84**	**71%**	**q=0.001*** **	
Entorhinal cortex	-0.29	8%	q=0.100	
Parahippocampal gyrus	-0.19	4%	q=0.100	
Fusiform gyrus	-0.57	32%	q=0.147	
Precuneus	-0.52	27%	q=0.229	
Superior parietal gyrus	-0.33	11%	q=0.978	
Inferior parietal gyrus	-0.71	50%	q=0.094	
Occipital cortex (V1)	-0.41	17%	q=0.530	

The table shows the association of cortical thickness with the p-tau load in regions of the right hemisphere in Alzheimer's disease. The columns show the correlation coefficient (r), explained variance in cortical thickness by p-tau load (R^2) and q-value (false discovery rate corrected p-value) of the correlations. In the right column, the regional correlation coefficient values (r) indicated in the second column are visually shown on the cortical surface. The table shows that the middle and superior frontal gyrus and the middle temporal gyrus hosted strong, significant negative associations between p-tau load and cortical thickness.

Supplementary Table 10. Correlations between post-mortem *in-situ* and ante-mortem *in-vivo* cortical thickness in Alzheimer's disease cases.

Case number	AD phenotype	Interval (years)	Interval (months)	r	p-value
**25**	Atypical (B/D)	<1	<1	0.95	p<0.001
**20**	Typical	<1	1	0.97	p<0.001
**27**	Atypical (B/D)	<1	3	0.97	p<0.001
**21**	Atypical (PCA)	1	17	0.95	p<0.001
**20**	Typical	2	22	0.98	p<0.001
**28**	Atypical (B/D)	2	24	0.89	p<0.001
**12**	Typical	2	26	0.96	p<0.001
**14**	Typical	2	28	0.96	p<0.001
**19**	Typical	2	28	0.86	p<0.001
**20**	Typical	3	30	0.94	p<0.001
**16**	Typical	4	48	0.91	p<0.001
**29**	Atypical (B/D)	4	50	0.87	p<0.001
**19**	Typical	4	52	0.87	p<0.001
**26**	Atypical (PCA)	5	54	0.95	p<0.001
**12**	Typical	5	60	0.88	p<0.001
**24**	Atypical (PCA)	5	63	0.73	p<0.001
**18**	Typical	6	72	0.94	p<0.001
**14**	Typical	7	82	0.92	p<0.001
**18**	Typical	7	89	0.90	p<0.001
**17**	Typical	10	120	0.69	p=0.001

The table shows the correlations between cortical thickness acquired post-mortem (and used for this study) and ante-mortem at different time intervals from death, measured both in months and years. Case numbers match those in Supplementary Table 1. Legend: AD = Alzheimer's disease; B/D = behavioral / dysexecutive variant; PCA = posterior cortical atrophy; r = correlation coefficient.

Supplementary figures

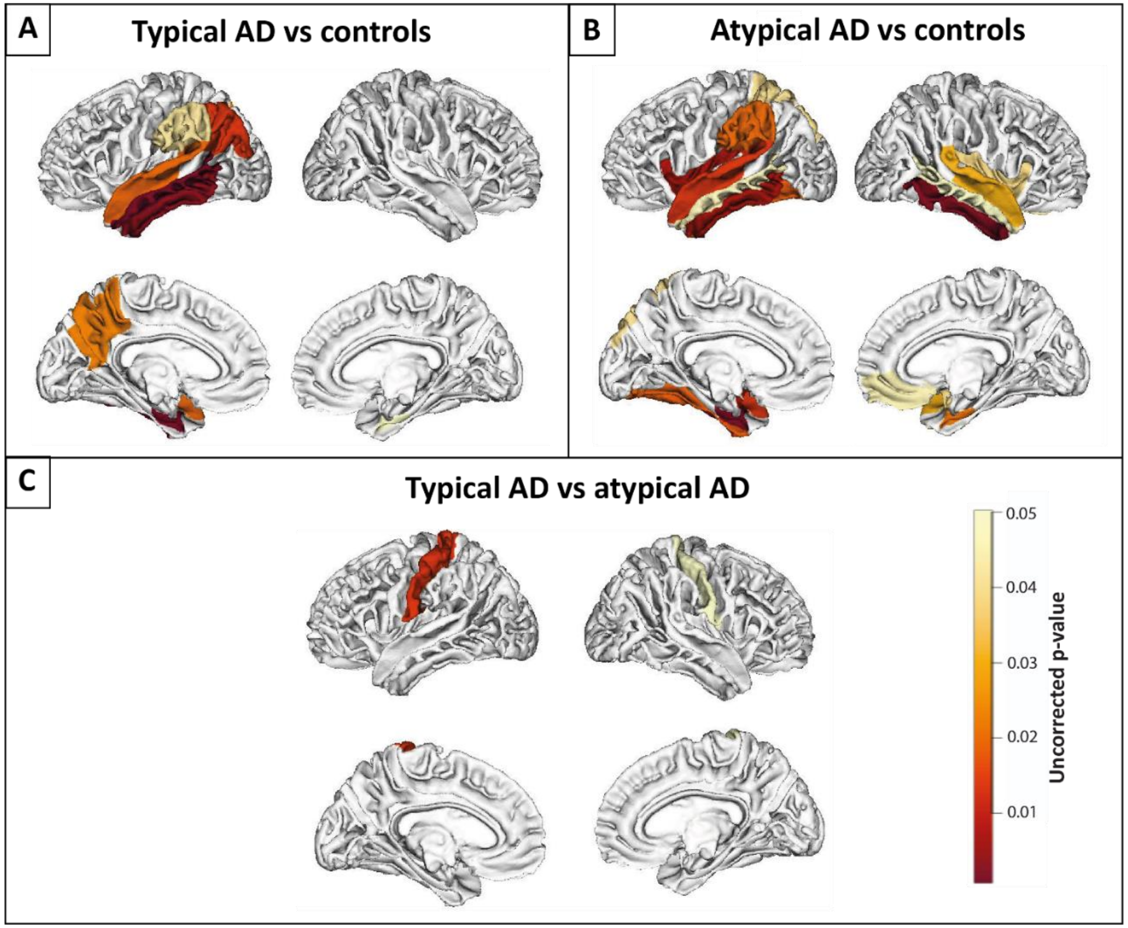

Supplementary Figure 1. Qualitative MRI cortical atrophy in typical and atypical Alzheimer's disease. No regional cortical thickness differences that survived FDR correction were found between controls, typical and atypical AD. In this figure we show the qualitative regional cortical thickness differences (the uncorrected p-values) in **A)** typical Alzheimer's disease (AD) versus controls, **B)** atypical AD versus controls,

and **C)** typical versus atypical AD across the whole cortex. From this figure we can see that typical AD had more lateralized atrophy (left hemisphere), while the cortical atrophy in atypical was present in both left and right hemisphere. The only regions that seemed to slightly differ between AD phenotypes were the left and right postcentral gyrus. The scale bar represents the uncorrected p-values. For detailed information, see Supplementary Table 5.

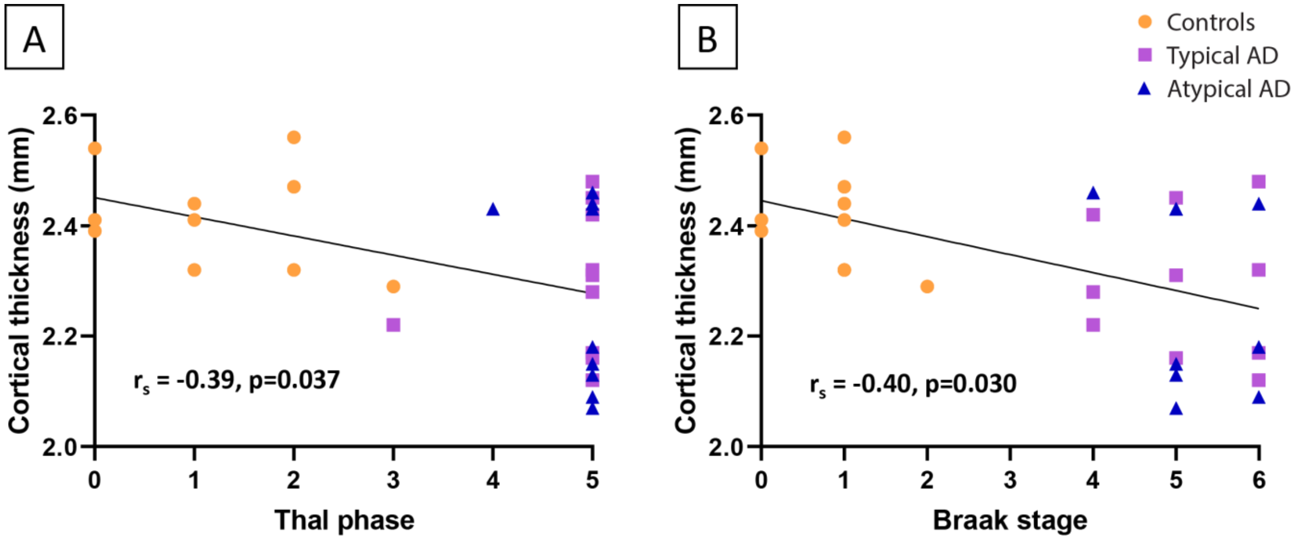

Supplementary Figure 2. Association of MRI cortical thickness with Thal phase (A) and Braak NFT stage (B) at autopsy in Alzheimer's disease. Averaged whole-brain cortical atrophy associated with a higher Thal phase[4] and Braak NFT stage[5] in the whole cohort, including controls, typical and atypical Alzheimer's disease. Legend: AD = Alzheimer's disease.

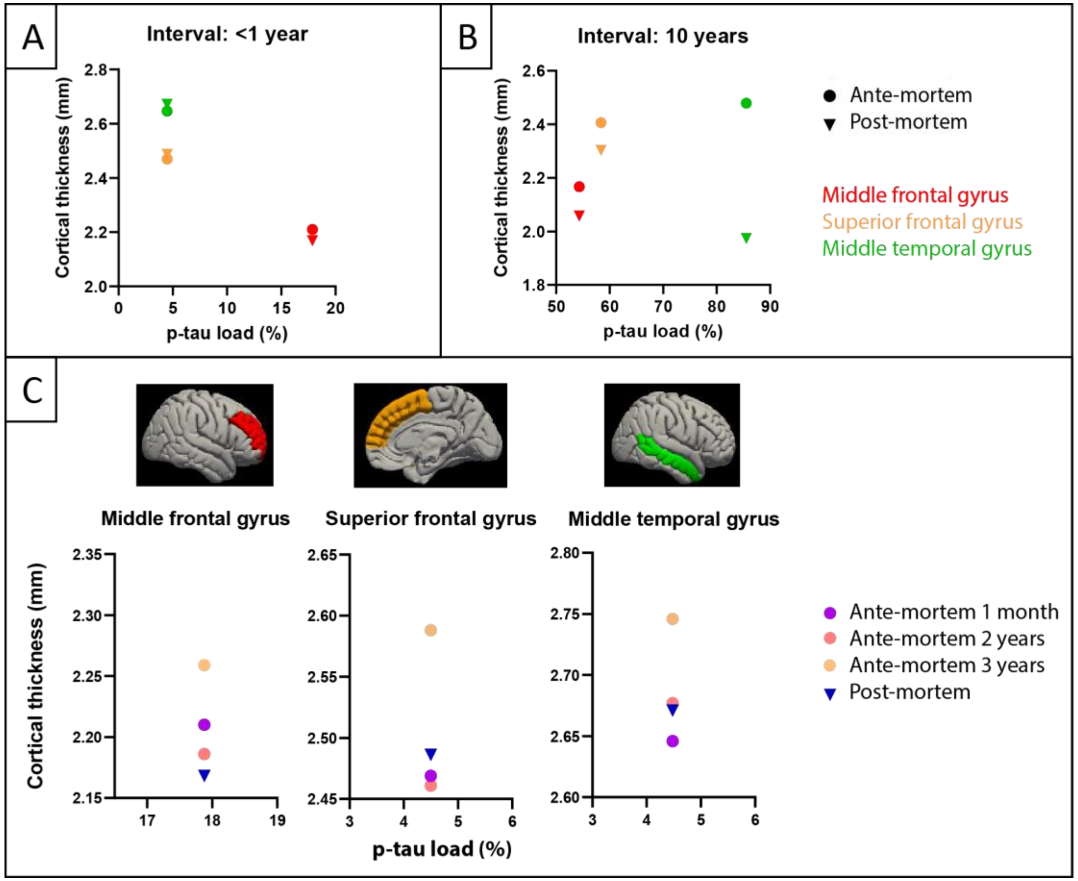

Supplementary Figure 3. Association of post-mortem and ante-mortem MRI cortical thickness with p-tau load in Alzheimer's disease. (A) and (B) show the association of p-tau load and both ante-mortem and post-mortem cortical thickness in the regions that hosted a significant association (see Paragraph 3.5). Particularly, (A) shows that the association between ante-mortem and post-mortem cortical thickness with p-tau load does not differ when the ante-mortem scan is acquired less than one year before death (case number 20 in Table S9), (B) while some variation is found when the scan is acquired 10 years before death, particularly in the middle temporal gyrus (case number 17 in Table S9). (C) shows a single AD case (case number 20 in Table S9) with three ante-mortem scans acquired at one-month (purple circle), two-years (pink circle), and three-years intervals (orange

circle) prior to death, compared to the post-mortem scan (blue triangle). In each brain area, the three-year ante-mortem scan showed the most pronounced difference in cortical thickness compared to the post-mortem scan.
